# Loss of AT8 Nuclear Tau as a Marker of Neuronal Ageing and Alzheimer’s Disease Progression

**DOI:** 10.3390/biomedicines13112587

**Published:** 2025-10-23

**Authors:** Francesca Bruno, Laura Gil, Valentina Sturiale, Carmen Guerrero, Ana Belen Rebolledo, Desiree Brancato, Javier Morales, Salvatore Saccone, Concetta Federico

**Affiliations:** 1 Department of Biological, Geological and Environmental Sciences, University of Catania, 95123 Catania, Italy; francesca.bruno@unikore.it (F.B.); valentina.sturiale@phd.unict.it (V.S.); desiree.brancato@phd.unict.it (D.B.); federico@unict.it (C.F.); 2 Department of Medicine and Surgery, Kore University of Enna, 94100 Enna, Italy; 3 Facultad de Medicina, Universidad Alfonso X el Sabio (UAX), 28691 Villanueva de la Cañada, Spain; lgilalb@uax.es (L.G.); jmoraper@uax.es (J.M.); 4 Banco de Cerebros (Biobanco), Hospital Universitario Fundación Alcorcón, 28922 Alcorcón, Spain; mcarmen.guerrero@salud.madrid.org (C.G.); anabelenr@fhalcorcon.es (A.B.R.)

**Keywords:** nuclear tau protein, Alzheimer’s disease, hippocampus, cell cycle re-entry, AT8 epitope, nucleolus, neuroblastoma cell line, human brain, neuronal differentiation

## Abstract

**Background/Objectives:** Tau protein, a central player in Alzheimer’s disease (AD) pathology, is classically known for its role in microtubule stabilisation. However, accumulating evidence indicates that tau also localises to the neuronal nucleus, particularly the nucleolus, where it may regulate chromatin organisation and transcription. In this study, we investigated whether different phosphorylation states of nuclear tau display age- and disease-dependent patterns, with a specific focus on the AT8 epitope (phospho-Ser202/Thr205). **Methods:** We analysed nuclear tau epitopes (Tau-1, AT8, PHF1, T181, and S262) by indirect immunofluorescence in SK-N-BE neuroblastoma cells under proliferative and retinoic acid-induced differentiated conditions and in post-mortem hippocampal CA1 neurons from foetal, young, aged, and AD brains. Other functional markers (UBTF, Ki67, fibrillarin and acetylated histone H4) were used to assess nuclear organisation and function. **Results:** Compared with the other epitopes, AT8 was unique in showing dynamic nuclear localisation: absent in proliferating cells but present after differentiation, abundant in young neurons, and significantly reduced in aged and AD samples. Nuclear AT8 co-localised with Ki67, and its decline was associated with neuronal cell cycle re-entry and nucleolar disorganisation. **Conclusions:** Among multiple nuclear tau epitopes, AT8 was the only one displaying age- and disease-related changes, and its reduction during ageing and AD correlates with nuclear stress, aberrant cell cycle activity, and neuronal vulnerability. Loss of nuclear AT8 may therefore represent an early marker of dysfunction in ageing and AD brains.

## 1. Introduction

Neurodegenerative disorders represent a heterogeneous group of chronic and progressive conditions affecting the central nervous system (CNS), in which specific neuronal populations gradually lose their function and viability. Among them, Alzheimer’s disease (AD) is the most prevalent and a major cause of dementia worldwide. A central pathological feature of AD is the abnormal accumulation of misfolded proteins, including β-amyloid (Aβ) and hyperphosphorylated tau, which contribute to synaptic dysfunction and neuronal loss. Despite decades of research, the molecular mechanisms that initiate and sustain these processes remain incompletely understood [1,2,3,4]. Among the various hypotheses proposed in the literature regarding the etiopathogenesis of neurodegenerative disorders, substantial evidence accumulated over the last two decades has supported the “cell cycle hypothesis,” which suggests that aberrant re-entry of post-mitotic neurons into the cell cycle represents an early and critical event in AD. This dysregulation is closely linked to tau phosphorylation and nuclear tau depletion, leading to chromatin disorganisation and nucleolar dysfunction [5,6,7,8,9,10,11,12,13].

In AD, cell cycle alterations in vulnerable neurons appear to contribute to the accumulation of hyperphosphorylated tau, leading to the formation of neurofibrillary tangles (NFTs) and the simultaneous expression of cell cycle markers. Specifically, tau phosphorylated at the AT8 epitope (Ser202/Thr205) localises to nuclear compartments such as the nucleolus and pericentromeric heterochromatin, where it may influence chromatin structure and transcriptional regulation [12,14,15,16]. In vitro and in vivo studies indicate that nuclear tau phosphorylation at epitopes including AT8, Tau-1, T181, and PHF1 correlates with neuronal differentiation states and nucleolar activity, suggesting a regulatory role in rDNA transcription and chromatin maintenance [14,17,18,19,20]. Dysregulation of this phosphorylation pattern has been associated with aberrant cell cycle re-entry and neuronal vulnerability in AD [15,17,21]. Several studies have specifically demonstrated the presence of Ki67 and cyclins in post-mitotic neurons of aged and AD brains [22,23,24], supporting the aberrant cell cycle re-entry hypothesis in neurodegeneration. Neuronal cell cycle re-entry can be either “abortive,” where neurons die at the G1/S checkpoint, or “non-abortive,” in which post-mitotic neurons undergo DNA synthesis in the S phase but fail to complete mitosis, ultimately dying before the G2/M transition [25,26,27,28]. It has been proposed that nuclear tau depletion is associated with chromatin instability at the nuclear lamina and nucleolus, along with dysfunctional nucleocytoplasmic reorganisation [14,17,29,30,31,32,33,34,35].

Intracellular NFTs and extracellular amyloid-β (Aβ) plaques are the main histopathological markers of AD, but their link to cognitive decline remains debated. While senile plaques are weakly correlated with cognitive symptoms, hyperphosphorylated tau is more strongly associated with synaptic dysfunction and disease progression [36,37]. The hippocampus, particularly CA1 neurons, is highly susceptible to tau pathology and neurodegeneration, making it a critical region for studying nuclear tau dynamics across ageing and disease progression [38,39,40,41,42,43,44,45,46,47]. However, the precise mechanisms by which nuclear tau phosphorylation contributes to neuronal stability, nucleolar homeostasis, and cell cycle control remain poorly defined.

While cytoplasmic NFTs are well-established hallmarks of AD and are closely associated with disease progression, recent findings indicate that nuclear tau localisation plays a critical role in the early stages of AD. Specifically, nuclear tau has been detected in two primary locations: the nucleolus and pericentromeric heterochromatin [34,38,48,49,50,51]. In vitro studies have identified specific nuclear tau epitopes, such as Tau-1 (Pro189/Gly207) and AT8 (pSer202/Thr205), which may interact directly with DNA and/or RNA. These epitopes appear to be involved in early AD events, potentially linking nuclear tau to neuronal differentiation. Notably, AT8 is considered a marker of neuronal differentiation, and its nucleolar localisation suggests an association with rDNA transcriptional repression [18,19,52,53].

In the present study, we focus on the nuclear functions of tau and its phosphorylation states, analysing neuroblastoma cells and human hippocampal neurons across different ages and AD stages. Our aim is to elucidate how tau phosphorylation at specific nuclear epitopes correlates with nucleolar integrity, cell cycle re-entry, and neurodegenerative processes. Understanding these nuclear tau dynamics could provide novel insights into early AD pathogenesis and highlight potential therapeutic targets.

## 2. Materials and Methods

### 2.1. Cell Cultures

The human neuroblastoma cell line SK-N-BE [54] was grown at 37 °C and 5% CO_2_ in RPMI 1640 supplemented with 10% foetal bovine serum (FBS) and 1% penicillin/streptomycin (100 U/mL; 100 μg/mL). The cells were differentiated into neuronal-like cells with 10 μM retinoic acid (RA) (Sigma–Aldrich, Darmstadt, Germany, Cat. No. R2625) as previously described [55,56]. The cell line used in this study is a well-established model, routinely employed in our laboratories, and extensively characterised in previous studies also by our group [53,55]. The differentiation status of the cells was carefully monitored during RA exposure, with neuronal-like features becoming evident within a few days of treatment (see Supplementary Figure S1).

### 2.2. Antibodies for Indirect Immunofluorescence

Immunodetection of specific antigens was performed via the following primary antibodies: Tau-1 (Millipore, Single Oak Dr, Temecula, CA, USA, Cat. No. MAB3420; dilution 1:100) for unphosphorylated tau (Pro189/Gly207); AT8 (Thermo Scientific, Rockford, IL, USA, Cat. No. MN1020; dilution 1:50) for pSer202/Thr205 tau; PHF-1 (Invitrogen, Rockford, IL, USA, Cat. No. 44-758G, dilution 1:100) for pSer396/Ser404 tau; T181 (Invitrogen, Rockford, IL, USA, Cat. No. MN1050; dilution 1:100) for pThr181 tau; S262 (Invitrogen, Rockford, IL, USA, Cat. No. 44-750G; dilution 1:100) for pSer262 tau; Ki67 (Invitrogen, Rockford, IL, USA, Cat. No. MA5-14520; dilution 1:100) as a cell proliferation marker; UBTF (Novus Biologicals, Briarwood Avenue, Centennial, CO, USA, Cat. N. NBP1-82545; dilution 1:100) to highlight the nucleolus; anti-fibrillarin (Clone 38F3; Invitrogen, Rockford, IL, USA, Cat. No. MA1-22000; dilution 1:800) for nucleolar activity; and anti-acetylated histone H4 (H4Ac, Santa Cruz Biotechnology, Dallas, TX, USA, Cat. No. sc-377520; dilution 1:50) for nuclear chromatin status. The secondary antibodies used included FITC-conjugated anti-mouse (Sigma–Aldrich, Saint Louis, MO, USA, Cat. No. F6257; dilution 1:300), FITC-conjugated anti-rabbit (Sigma–Aldrich, Saint Louis, Missouri, USA, Cat. No. F4890; dilution 1:100), and TRITC-conjugated anti-rabbit (Sigma–Aldrich, Saint Louis, Missouri, USA, Cat. No. T6778; dilution 1:400) antibodies. While some antibodies have been reported to be non-specific [57], this was excluded because the antibodies were validated by Federico et al. (2018) [53] through Western blot analyses, including the TAU-5 antibody (total tau).

### 2.3. Immunodetection on the Neuroblastoma Cell Line

Indirect immunofluorescence (IIF) experiments were performed as previously described [53]. Briefly, SK-N-BE cells were cultured on glass chamber slides, fixed with 4% paraformaldehyde for 20 min at room temperature, and permeabilised with 0.5% Triton X-100 (Chemsolute, Hamburg, Germany, Cat. No. 8059.0500). Immunodetection was carried out by overnight incubation with the specific primary antibody, followed by incubation with the corresponding secondary antibody. The cell nuclei were stained with DAPI (blue). Images were acquired via a confocal laser scanning microscope (CLSM) (LSM700, Zeiss, Oberkochen, Baden-Württemberg, Germany) equipped with 40× and 63× objectives and ZEN 2010 software.

### 2.4. Tissue Sections

Human brain sections were obtained in an anonymised form from the Tissue Biobank of the Hospital Universitario Fundación Alcorcón (HUFA), C/Budapest 1, 28922 Alcorcón, Madrid, Spain. All experiments were performed in accordance with the Declaration of Helsinki and approved by the Clinical Research Ethics Committee of Hospital Universitario Fundación Alcorcón (no. 23/69, on 14 June 2023). The brain tissue sections, which were prepared as previously described [30,58], were obtained from the *Cornu Ammonis 1* (CA1) region of foetuses, young, and elderly individuals with no Alzheimer’s disease (AD) diagnosis (Supplementary Table S1). These subjects had not received previous radiation or chemotherapy. Brain tissues from individuals with a post-mortem diagnosis of AD were classified into severity grades (AD-I to AD-IV) according to Braak’s classification [43]. Pyramidal neurons in the CA1 region of the hippocampus were identified based on morphological criteria assessed by DAPI staining, including: (i) localisation within the pyramidal layer, (ii) large euchromatin-rich nuclei, and (iii) a prominent, centrally positioned nucleolus (Supplementary Figure S2). These features reliably distinguish neurons from glial cells, which exhibit smaller nuclei with dense heterochromatin and lack a distinct nucleolus [59].

### 2.5. Immunodetection on Human Tissue Sections and CLSM Analyses

Paraffin-embedded sections for IIF experiments were processed as previously described [60,61]. Briefly, human tissue sections were deparaffinised in xylene, rehydrated in graded alcohols, and treated with citrate buffer and Sudan Black to reduce autofluorescence. The samples were incubated overnight at 4 °C with a specific primary antibody, followed by incubation with a secondary antibody and staining with DAPI. Each IIF experiment was repeated at least three times. Images were acquired via CLSM (LSM700, Zeiss, Oberkochen, Baden-Württemberg, Germany) equipped with 40× and 63× objectives and ZEN 2010 software for image acquisition.

### 2.6. Cell Counting and Statistical Analysis

Cell counting for statistical analyses was performed as previously described [58,62]. Specifically, IIF-positive cells were counted in 0.5-μm scanned images acquired via CLSM. The number and percentage of cells showing specific IIF signals were determined in six optical fields at 400× magnification per experiment. The quantitative detection of the AT8 epitope was performed via fluorescence intensity profiles using ZEN-2010 software.

Statistical analyses were conducted via Prism v. 8.0 (GraphPad Software, San Diego, CA, USA). Normality was tested with the Kolmogorov–Smirnov test, and significant differences between pairs of groups (epitope-positive cells vs. controls) were assessed via Student’s *t*-test. The data are presented as the mean ± standard error of the mean (S.E.M.), with significance levels set at *p* < 0.05 (*), *p* < 0.01 (**), *p* < 0.001 (***), and *p* < 0.0001 (****).

## 3. Results

### 3.1. Tau Epitopes in the Nucleus of Neurons

The tau protein contains multiple phosphorylatable sites, giving rise to various epitopes. We analysed the AT8 and Tau-1 epitopes, representing the unphosphorylated (Tau-1) and the phosphorylated (AT8) condition of the same protein region, Pro189/Gly207 and pSer202/Thr205, respectively, and the PHF1, T181, and S262 epitopes, which correspond to hyperphosphorylated tau, tau phosphorylated at threonine 181, and tau phosphorylated at serine 262, respectively. IIF was performed on neuroblastoma cells (replicative and neuronal-like differentiated conditions), and in CA1 brain tissue sections from subjects of different ages and at different AD stages.

#### 3.1.1. Nuclear Tau Epitopes in SK-N-BE Neuroblastoma Cell Line

**Figure 1:**
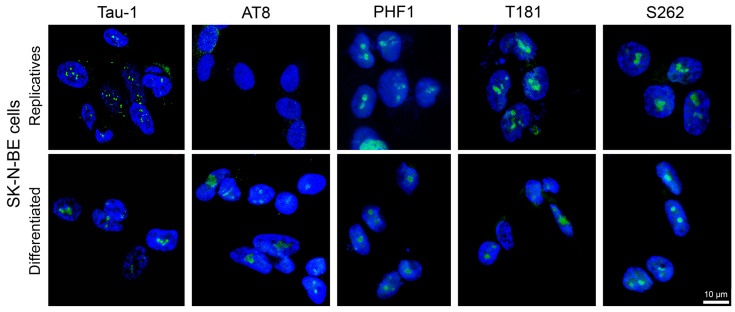
Detection of tau epitopes in SK-N-BE cells. Immunodetection of Tau-1, AT8, PHF1, T181, and S262 epitopes (green signals) was obtained in replicating (**upper images**) and RA-differentiated cells (**bottom images**). Nuclei were counterstained with DAPI (blue). Scale bar: 10 µm (bottom right corner; applies to all images). Separated green and blue channels are shown in Supplementary Figure S3.

In untreated replicating SK-N-BE cells, the Tau-1, PHF1, T181, and S262 epitopes were detected in the nucleolus, whereas AT8 was absent (Figure 1, upper images). However, in cells differentiated with retinoic acid (Supplementary Figure S1), all analysed epitopes were observed in the nucleolus (Figure 1, bottom images). For Tau-1 and AT8, the present results are consistent with previous findings [53].

**Figure 2:**
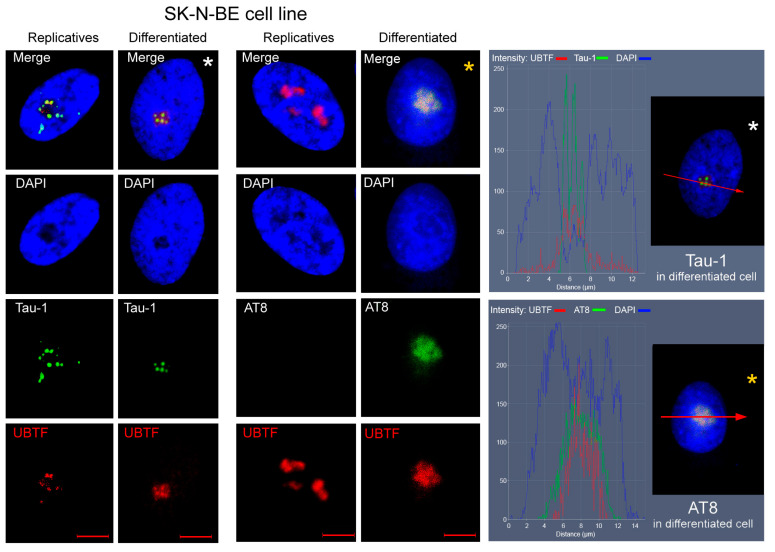
Co-localisation of tau epitopes and UBTF in the nuclei of SK-N-BE cells. The Tau-1 and AT8 epitopes (green signals) were co-localised with the nucleolar marker UBTF (red signals) in the nucleoli of replicating and RA-differentiated SK-N-BE cells. The AT8 epitope was absent in untreated replicating cells. Nuclei were counterstained with DAPI (blue). Scale bars: 5 µm. On the right, the nuclei of RA-differentiated cells (indicated by white and yellow asterisks) were analysed using fluorescence intensity profiles, clearly showing the overlap of red (UBTF) and green (Tau-1 or AT8) signals. Red arrows indicate the position of the fluorescence intensity profile along the nucleus, as shown in the adjacent intensity plot.

The nucleolar localisation of tau epitopes was further confirmed by co-localisation of AT8 and Tau-1 with the nucleolar marker upstream binding transcription factor (UBTF) (Figure 2). Tau-1 and AT8 were present in nearly all nucleoli, with fewer than 5% of nuclei lacking detectable signal.

Thus, among the five nuclear tau epitopes analysed, AT8 was the only one showing a clear presence/absence pattern between differentiated and replicative SK-N-BE cells.

#### 3.1.2. Nuclear Tau Epitopes in Human Neurons from the CA1 Region of the Hippocampus

**Figure 3:**
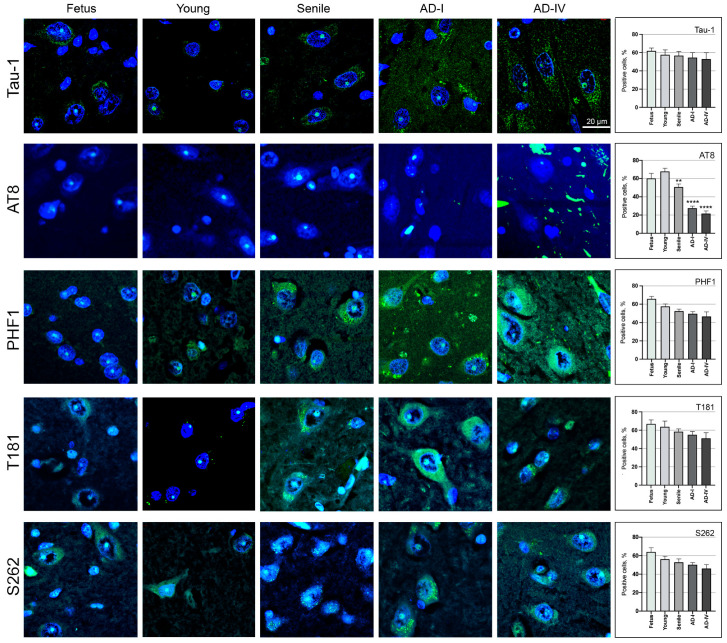
Detection of tau epitopes in neurons from the human CA1 region. Representative IIF images showing the detection of the Tau-1, AT8, PHF1, T181, and S262 epitopes (green) in brain sections from the CA1 region of the hippocampus. Nuclei were stained with DAPI (blue). For each epitope, representative images are presented from left to right, showing tissue sections from normal subjects (foetus, young and senile) as well as from subjects with AD at stages AD-I and AD-IV. The scale bar, located in the bottom right corner of the AD-IV section in the Tau-1 row, applies to all images. The graphs on the right of each row depict the percentage of neuronal cells positive for the respective epitope across different ages and AD progression stages. Statistically significant differences for the AT8 epitope, with respect to the young sample, are indicated by ** (*p* < 0.01), **** (*p* < 0.0001). In all the other cases, non-significant differences compared to the young brain are not shown.

The nuclear localisation of the Tau-1, AT8, PHF1, T181, and S262 epitopes was analysed in CA1 neurons in brain sections from foetal, young, and aged individuals, as well as from AD patients at stages I and IV of disease progression (Figure 3). We observed the presence of all epitopes in the nucleus of CA1 neurons with an age-dependent decrease in the percentage of cells positive for the analysed epitopes. More precisely, Tau-1, PHF1, T181, and S262 epitopes showed a gradual decrease in the percentage of positive cells from foetus to senile ages and AD stages. No statistically significant differences were detected when compared with the young brain.

For the AT8 epitope, we observed the highest percentage of AT8-positive neurons in young subjects. Compared to this condition, the percentage significantly decreased in aged samples (*p* < 0.01) and even more markedly in AD samples (*p* < 0.0001). No statistically significant differences were detected for Tau-1, PHF1, T181, or S262 in any group compared to the young brain (Figure 3). A summary table (Supplementary Table S2) provides a comparative overview of AT8, Tau-1, PHF1, T181, and S262 nuclear positivity in SK-N-BE cells (proliferative and differentiated) and in human hippocampal tissue across different ages and AD stages.

### 3.2. AT8 Epitope in Human Neurons from the CA1 Region of the Hippocampus

**Figure 4:**
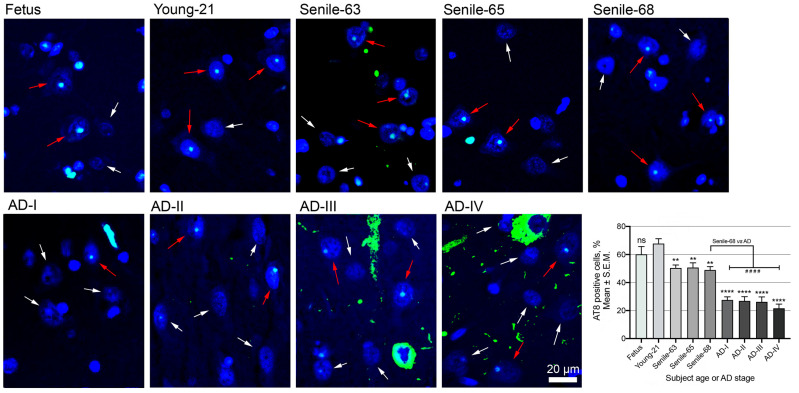
AT8 epitope detection in neurons from the human CA1 region. Each panel shows the immunofluorescence detection of the AT8 epitope (green signals) in brain sections from the CA1 region of the hippocampus. Cell nuclei are counterstained with DAPI (blue). The upper row displays tissue sections from individuals at different ages, ranging from foetal to senile, while the lower row presents sections from subjects with AD at different stages of progression (AD-I to AD-IV). Red arrows indicate nucleolar AT8-positive neurons, whereas white arrows point to nuclei lacking AT8 signals or with levels below the detection threshold. The scale bar, common to all images, is shown in the bottom right corner of the AD-IV panel. The ages of the “young” and “senile” subjects are indicated next to each label. The lower right portion of the figure displays a graph showing the percentage of AT8-positive neurons across different ages and AD stages. Statistical significance is indicated by ** and ****, corresponding to *p* < 0.01 and *p* < 0.0001, respectively, in comparisons with the young subject (used as reference). The symbol #### indicates statistically significant differences (*p* < 0.0001) between AD cases and the senile-68 subject, used as a reference to assess differences between aged and AD samples. ns indicates not significant.

Considering the unique behaviour of AT8 compared with the other analysed epitopes, both in neuroblastoma cells (Figure 1 and Figure 2) and in CA1 brain sections (Figure 3), we further investigated its presence in CA1 neurons in more detail, including stages II and III of AD (Figure 4). Quantitative analysis confirmed that the percentage of AT8-positive neurons in AD brains was markedly reduced (*p* < 0.0001) to ~26% in AD samples, compared to ~68% in young and ~50% in senile individuals. No significant differences were observed among the three senile samples or among the four AD stages. All elderly samples showed a significant reduction (*p* < 0.01) compared to young samples, and all AD stages confirmed the strongest reduction (*p* < 0.0001) compared with both young and senile individuals (Figure 4).

**Figure 5:**
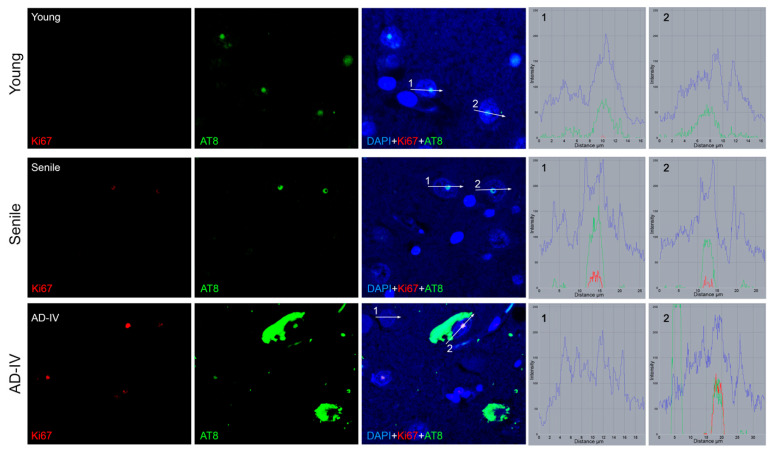
Co-detection of Ki67 and AT8 in neurons from the human CA1 region. Representative images from young, senile and AD-IV brain sections are shown (**top** to **bottom**). For each sample, Ki67 (red), AT8 (green), and the merged signals (with DAPI, blue) are displayed. On the right, fluorescence intensity profiles were generated using ZEN2010 Software (Zeiss) along the white line across indicated cells (cell no. 1, and 2 in each image). In the AD-IV image, neurofibrillary tangles are visible as large green aggregates, and one of these cells also shows a high level of Ki67 (red profile). The different colors of the intensity profiles correspond to AT8 (green), Ki67 (red), and DAPI (blue).

Brain sections from individuals of different ages and at various stages of AD progression were analysed for the presence of cells with an active cell cycle using Ki67 as a marker. Specifically, Ki67 and the AT8 epitope of nuclear tau were co-detected by IIF (Figure 5). The results revealed a highly significant increase in the number of Ki67-positive cells in senile and AD brain sections compared to young subjects (*p* < 0.0001). In young brain sections, Ki67-positive neurons were rare (less than 3%), whereas their frequency increased in senile (23%) and AD (38%) brain sections.

Beyond the variation in the number of Ki67-positive cells, differences were also observed in Ki67 signal intensity within individual cells (Figure 5). In young neurons, Ki67 was detected at very low levels (<10 fluorescence intensity units, measured with ZEN2010 software), while AT8 intensity was consistently higher (>50). In senile neurons, Ki67 intensity increased (>20), along with an even higher AT8 signal (>80). In AD neurons, Ki67 reached its highest levels (>50), becoming comparable to AT8 intensity. Importantly, all Ki67-positive neurons also exhibited nuclear AT8 immunoreactivity, and no cells displayed Ki67 signal in the absence of AT8, suggesting a strict association between aberrant cell cycle re-entry and AT8-positive tau in the nucleus.

### 3.3. Transcriptional Status of Neuronal Cells in the CA1 Brain Region

**Figure 6:**
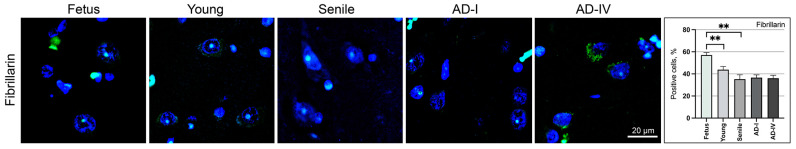
Detection of fibrillarin in neurons from the CA1 region at different ages and stages of AD progression. Representative IIF images showing fibrillarin (green signals) in brain sections from the CA1 region of the hippocampus. The cell nuclei were counterstained with DAPI (blue). From left to right, images correspond to tissue sections from normal subjects (foetal, young, and senile), and subjects with AD at stages AD-I and AD-IV. The scale bar in the bottom right corner of the AD-IV image applies to all panels. The graph on the right displays the percentage of fibrillarin-positive neuronal cells, with statistically significant differences indicated (**, *p* < 0.05). In other cases, the differences were not statistically significant.

The transcriptional status of neurons in the CA1 region was assessed through the detection of fibrillarin and acetylated histone H4 (H4Ac) in the nucleus. The results revealed a gradual and statistically significant age-related decrease in fibrillarin levels in the nucleolus of neurons. However, no significant differences were observed between senile and AD subjects (Figure 6).

**Figure 7:**
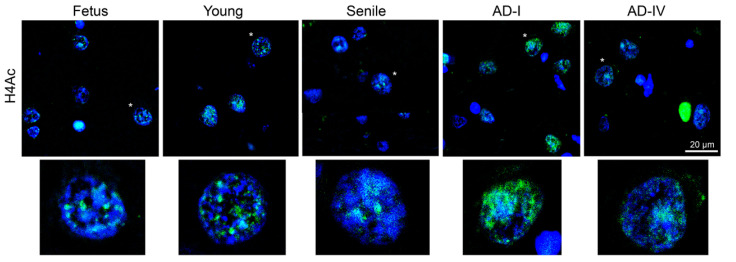
Detection of acetylated histone H4 (H4Ac) in neurons from the human CA1 region at different ages and stages of AD progression. Representative IIF images (**upper panel**) showing the H4Ac detection (green signals) in brain sections from the CA1 region of the hippocampus. The cell nuclei were counterstained with DAPI (blue). From left to right, tissue sections correspond to normal subjects (foetal, young, and senile), and subjects with AD at stages AD-I and AD-IV. The scale bar in the bottom right corner of the AD-IV image applies to all panels. Statistical analysis of the number of H4Ac-positive neurons revealed no significant differences among different ages or AD stages. The lower panel shows magnified images of the nuclei marked with a white asterisk in the corresponding upper images, highlighting differences in H4Ac distribution.

With respect to H4Ac detection, the number of H4Ac-positive cells did not differ significantly across groups. However, notable variations were observed in the nuclear distribution of H4Ac: in foetal and young neurons, the signal was confined to relaxed chromatin regions, whereas in senile and AD neurons, it appeared more diffusely distributed throughout the nucleus (Figure 7).

## 4. Discussion

Extensive knowledge exists concerning the cellular and tissue changes that occur in the hippocampal region and are associated with Alzheimer’s disease (AD), such as the accumulation of neurofibrillary tangles with hyperphosphorylated tau protein and beta-amyloid deposits [3,37]. However, the initial triggers of neurodegeneration in AD remain elusive. The tau protein and its post-translational modifications, particularly hyperphosphorylation, play significant roles in neuronal dysfunction and death. The amount and type of neurofibrillary tangles observable in the hippocampus are key indicators of disease progression [17,43].

Tau, encoded by the microtubule-associated protein tau (MAPT) gene, is known primarily for stabilising microtubules, but increasing evidence suggests that it also has nuclear functions in chromatin organisation and protection [14,19,58,63]. Among the nuclear epitopes of tau, Tau-1 and AT8 are particularly relevant for its interaction with DNA. These epitopes, which mark the same protein region but in different phosphorylation states, are linked to structural changes that could influence the chromatin-binding properties of tau. AT8 phosphorylation at Ser202/Thr205 is particularly important for nuclear tau function, suggesting roles in maintaining nuclear integrity and transcriptional regulation [14,49,53,64].

While AT8 shows marked and progressive nuclear changes with ageing and AD, other phosphorylated tau epitopes such as T181, S262, and PHF1 also localise to the nucleus and may regulate nuclear processes. This suggests that nuclear tau regulation and involvement in cell cycle re-entry are controlled by a coordinated phosphorylation programme across multiple sites. Therefore, AT8 could serve as a key marker within a complex phosphorylation landscape modulating nuclear functions and neuronal vulnerability.

Present and previous data indicate that the AT8 epitope is absent in proliferative neuroblastoma cells but appears upon differentiation or actinomycin-D (Act-D) treatment, supporting its association with reduced transcriptional activity [53,62]. Other tau epitopes (Tau-1, PHF1, T181, and S262) do not exhibit this pattern, reinforcing the specificity of AT8 in the function of nuclear tau. Therefore, although differentiation and transcriptional blockade are mechanistically distinct, both conditions reduce nucleolar activity and may promote the accumulation or redistribution of tau within the nucleus. These observations support the hypothesis that nuclear tau responds dynamically to nucleolar stress or changes in transcriptional homeostasis. Although in the present study we compared proliferative and differentiated conditions as distinct experimental endpoints, a more detailed time-course analysis of SK-N-BE differentiation could provide valuable insights into the dynamics of tau nuclear localization. Future investigations should therefore include longitudinal differentiation studies to better capture the temporal progression of nuclear AT8 accumulation and redistribution.

In human pyramidal neurons of the hippocampal CA1 region across different ages and AD stages, we observed a significant decline in nucleolar AT8-positive neurons from youth to senility, with a further marked decrease in AD cases. This progressive loss parallels changes in fibrillarin and histone acetylation, indicating alterations in transcriptional regulation and chromatin organisation during ageing and neurodegeneration. AT8 phosphorylation at Ser202/Thr205 may influence chromatin structure by modulating tau’s interaction with nuclear proteins involved in transcriptional regulation, DNA repair, or nucleolar integrity, suggesting that specific nuclear partners of AT8-phosphorylated tau—such as fibrillarin, histone-modifying enzymes, and transcription factors—could mediate the observed effects on chromatin organisation and nucleolar function. Alternative or complementary mechanisms may also contribute to AT8 depletion, which are discussed in the following paragraph. While ageing is an important factor to consider when interpreting changes in AT8 localisation, our findings support the hypothesis that the alterations observed in AD are not solely age-related. Notably, the older control group (63–68 years old) did not exhibit a comparable depletion of nuclear AT8 to that observed in AD stages. It should be noted that the number of human post-mortem samples analysed in this study was limited, which may constrain the generalisation of the observed trends. Nevertheless, the consistency of AT8 nuclear depletion across independent specimens supports the robustness of these findings. Consistent with previous studies showing that ageing can predispose neurons to tau pathology by promoting chromatin relaxation and transcriptional instability [14,36], our data suggest that early AD stages may act synergistically with ageing to exacerbate the loss of nuclear AT8, a phenomenon that becomes more pronounced as the disease progresses. Collectively, these findings point to a disease-specific vulnerability that extends beyond chronological ageing and reflects a combination of age-related chromatin dysregulation and early AD-associated nuclear alterations, including aberrant reactivation of the cell cycle in post-mitotic neurons.

The correlation between AT8 loss and neuronal degeneration suggests that nuclear AT8 may play a protective role under physiological conditions. However, its depletion could predispose neurons to aberrant cell cycle re-entry. Previous studies have shown that neuroblastoma cells can be induced to re-enter the cell cycle upon exposure to forskolin or aniline, which coincides with the loss of nuclear AT8 and increased cyclin expression [62]. In mature neurons, which are post-mitotic, inappropriate reactivation of the cell cycle may result in cellular stress and contribute to neurodegeneration. Interestingly, in hippocampal tissue from AD patients, we identified Ki67-positive neurons that also retained nuclear AT8 staining, while other AT8-positive neurons were Ki67-negative. This distribution does not suggest a strictly inverse relationship between the two markers. Instead, it indicates that nuclear AT8 and cell cycle reactivation can coexist in vulnerable neurons. These observations suggest that while nuclear AT8 may support neuronal stability under normal conditions, its persistence during replicative stress may reflect a dysfunctional nuclear state that contributes to degeneration. Our co-localisation data align with previous reports describing ectopic expression of Ki67 and cyclins in post-mitotic neurons during neurodegeneration [6,22,23,24,65,66].

Importantly, neuroblastoma cells and mature neurons may differ in their response to proliferative stimuli and nuclear AT8 dynamics. While cell cycle reactivation in neuroblastoma cells typically correlates with decreased nuclear AT8, in aged or AD neurons, AT8 may persist despite the presence of proliferation markers. Alternative mechanisms could also contribute to the observed depletion of AT8 in vulnerable neurons. These include tau mislocalization to the cytoplasm, selective proteasomal degradation, or impaired nucleocytoplasmic transport dynamics. Within the nucleus, AT8-phosphorylated tau may transiently interact with nucleolar components, such as fibrillarin, nucleolin, or RNA-binding proteins, thereby influencing ribosomal biogenesis and the maintenance of nucleolar integrity. Disruption of these interactions, possibly coupled with altered phosphorylation turnover or stress-induced tau mislocalization, could compromise nucleolar homeostasis and contribute to neuronal vulnerability. Although AT8 is mainly detected within the nucleolus, its dynamic redistribution between nucleolar and nucleoplasmic compartments could indirectly influence chromatin organisation and transcriptional activity. This suggests that the role of nuclear AT8 phosphorylation is context-dependent and may reflect distinct cellular responses to stress and injury in proliferative versus terminally differentiated cells.

Together with the evidence of chromatin alterations, we observed progressive depletion of nucleolar fibrillarin and altered patterns of intranuclear histone H4 acetylation, consistent with transcriptional and chromatin reorganisation accompanying nuclear AT8 alterations. However, these results are correlative in nature and do not establish a direct mechanistic link between AT8 redistribution and nuclear remodelling. While quantification of fibrillarin and H4Ac provides indirect measures of transcriptional activity and chromatin state, further studies involving co-localisation and functional assays will be required to elucidate the precise role of nuclear tau in regulating nucleolar function and transcription during ageing and neurodegeneration.

Our results also suggest that nuclear tau phosphorylation at multiple epitopes—including AT8 (Ser202/Thr205), T181, S262, and S404 (PHF1)—may be associated with nucleolar activity, although AT8 shows the most consistent and dynamic changes. In particular, the consistent nucleolar localisation of these phosphoepitopes observed both in neuroblastoma cells under different conditions (proliferative, differentiated, and transcriptionally inhibited) and in hippocampal CA1 neurons across ageing and Alzheimer’s disease stages points to a potential role of phosphorylated tau in supporting nucleolar structure and function, possibly contributing to the maintenance of ribosomal biogenesis and transcriptional homeostasis.

Notably, the unique decline of AT8 nucleolar localisation across life stages and Alzheimer’s disease progression—distinct from other epitopes—highlights a potentially critical role of this phosphorylation event in maintaining nucleolar homeostasis. This suggests that the loss of nuclear AT8 may represent a key event marking the shift from physiological to pathological tau function in post-mitotic neurons. Moreover, the relatively stable nuclear presence of other phosphoepitopes may reflect less dynamic roles or reduced susceptibility to nuclear remodelling during ageing and neurodegeneration. Altogether, these findings reinforce the notion that phosphorylation of tau at the AT8 epitope is closely linked to neuronal cell cycle control mechanisms, and that its alteration may increase neuronal vulnerability and contribute to aberrant cell cycle re-entry observed in neurodegenerative conditions. Our findings align with recent evidence suggesting that tau may exert a protective effect against neurodegeneration by modulating the neuronal cell cycle [15].

Taken together, these correlative results support a model in which nuclear tau plays a key role in maintaining neuronal homeostasis through regulation of chromatin state and suppression of aberrant cell cycle activity. However, functional validation in model systems remains essential to confirm these mechanisms. Each experimental model used in this study has inherent limitations that should be considered when interpreting the results. The SK-N-BE neuroblastoma cell line provides a tractable system to study differentiation-related nuclear tau changes but does not fully reproduce mature neuronal physiology, while post-mortem hippocampal tissue is subject to inter-individual variability and fixation effects. However, when used in combination, these complementary models provide convergent evidence supporting the robustness of our findings and allow cross-validation between controlled in vitro conditions and human pathological contexts.

The gradual loss of nuclear tau and the associated changes in the chromatin state, including fibrillarin reduction and histone H4 acetylation redistribution, provide new insights into the early molecular events in AD. These findings suggest that neuronal chromatin disorganisation may precede classical hallmarks of neurodegeneration, reinforcing the idea that the role of tau in the nucleus should be further investigated as a potential therapeutic target. Future studies should test whether site-specific tau mutants (phospho-deficient or phospho-mimetic at S202/T205) affect nuclear localisation and neuronal stability in models like neuroblastoma cells. Additionally, identifying biomarkers of nuclear tau depletion in accessible fluids could enable early AD detection.

## 5. Conclusions

A critical aspect of Alzheimer’s disease pathogenesis is the identification of the initial molecular events that trigger neuronal degeneration. Our findings indicate that the disappearance of the AT8 epitope from the neuronal nucleus constitutes a key early event linked to neurodegeneration onset. This loss correlates with ectopic cell cycle reactivation, supporting the hypothesis that aberrant attempts to re-enter the cell cycle are a primary cause of neuronal death in AD. The observed reduction in fibrillarin and changes in histone H4 acetylation patterns suggest nuclear remodelling events that may accompany the loss of nuclear tau, potentially affecting transcriptional stability and chromatin organisation. Although our results are correlative in nature and based on a limited number of neuropathological samples, the integration of ex vivo human tissue with in vitro cellular models strengthens the reliability of our findings. While each model presents specific limitations, their coordinated use offers a significant advantage: in vitro experiments enable controlled mechanistic observations, whereas human post-mortem analyses provide pathological validation. The convergence of these approaches increases confidence in the observed relationship between nuclear tau depletion, chromatin remodelling, and neuronal vulnerability. Larger cohort studies will be essential to confirm these results and to further clarify the link between nuclear tau loss, chromatin remodelling, and neuronal vulnerability.

Furthermore, our data indicate that tau phosphorylation at different sites may underlie distinct nuclear roles. While multiple phosphoepitopes are stably retained in the nucleolus, the AT8 epitope (Ser202/Thr205) shows a progressive nuclear loss, emerging as a specific marker of chromatin vulnerability and neuronal stress in Alzheimer’s disease.

The identification of circulating biomarkers reflecting nuclear tau depletion could be a valuable tool for early detection of AD-related neurodegeneration. Therapeutic strategies aimed at preserving nuclear tau function may help maintain neuronal integrity and prevent disease progression. In vivo studies and analyses of peripheral biofluids could further validate these findings and determine whether nuclear tau phosphorylation patterns, particularly AT8, may serve as accessible biomarkers of early neuronal vulnerability. Future research should focus on defining the molecular partners of nuclear tau and on investigating interventions that stabilise its nuclear functions in ageing neurons. In addition, functional assays, such as transcriptional activity analyses, and transcriptomic or proteomic approaches could clarify the molecular pathways linking AT8 loss to cell cycle re-entry. Although most therapeutic strategies currently focus on cytoplasmic tau, some may also influence nuclear tau, particularly those targeting tau phosphorylation, offering valuable translational perspectives for disease-modifying interventions. Advanced cellular models and in vivo systems will be crucial to experimentally test these interventions and to assess their efficacy in preserving nuclear tau function and neuronal integrity. Moreover, a deeper investigation of tau phosphorylation dynamics within the nucleolus could reveal a critical molecular crossroad linking chromatin remodelling and cell cycle reactivation in AD. By targeting the earliest neuronal dysfunction events, preventive strategies could be developed to mitigate Alzheimer’s disease progression before substantial neurodegeneration occurs.

## Data Availability

The data are contained within the article and in the Supplementary Materials.

## Supplementary Materials

The following supporting information can be downloaded at: https://www.mdpi.com/article/10.3390/biomedicines13112587/s1, Table S1: Summary of the cases included in the study; Table S2: Comparative analysis of nuclear tau epitopes in SK-N-BE cells and human hippocampal neurons across ages and AD stages; Figure S1: Differentiation of SK-N-BE neuroblastoma cell lines upon retinoic acid (RA) treatment; Figure S2: Identification of pyramidal neurons in the CA1 region. Figure S3: Visualisation of the blue and green channels corresponding to the images shown in Figure 1.

## Abbreviations

The following abbreviations are used in this manuscript:

Act-D	Actinomycin-D
AD	Alzheimer’s disease
Aβ	Amyloid-β
CA	Cornu Ammonis
CLSM	Confocal laser scanning microscopy
CNS	Central nervous system
FBS	Foetal bovine serum
H4Ac	Acetylated histone H4
HUFA	Hospital Universitario Fundación Alcorcón
IIF	Indirect immunofluorescence
*MAPT*	Microtubule-associated protein tau
NFTs	Neurofibrillary tangles
RA	Retinoic acid
UBTF	Upstream binding transcription factor
