# Supplementary material for: Loss of AT8 Nuclear Tau as a Marker of Neuronal Ageing and Alzheimer’s Disease Progression
Source: Biomedicines. 2025 Oct 23;13(11):2587. doi: 10.3390/biomedicines13112587 (PMC12650037; doi:10.3390/biomedicines13112587)
Supplement: Supplementary file 1 [file biomedicines-13-02587-s001.zip › biomedicines-3902273-supplementary.pdf]

Loss of AT8 Nuclear Tau as a Marker of Neuronal Ageing and Alzheimer's Disease Progression

Francesca Bruno [1,2], Laura Gil [3], Valentina Sturiale [1], Carmen Guerrero [4], Ana Belen Rebolledo [4], Desiree Brancato [1], Javier Morales [3], Salvatore Saccone [1,*] and Concetta Federico [1]

[1] Department of Biological, Geological and Environmental Sciences, University of Catania, 95123 Catania, Italy; francesca.bruno@unikore.it (F.B.); valentina.sturiale@phd.unict.it (V.S.); desiree.brancato@phd.unict.it (D.B.); federico@unict.it (C.F.)
[2] Department of Medicine and Surgery, Kore University of Enna, 94100 Enna, Italy
[3] Facultad de Medicina, Universidad Alfonso X el Sabio (UAX), 28691 Villanueva de la Cañada, Spain; lgilalb@uax.es (L.G.); jmoraper@uax.es (J.M.)
[4] Banco de Cerebros (Biobanco), Hospital Universitario Fundación Alcorcón, 28922 Alcorcón, Spain; mcarmen.guerrero@salud.madrid.org (C.G.); anabelenr@fhalcorcon.es (A.B.R.)
* Correspondence: salvatore.saccone@unict.it

Supplementary Table S1: Summary of the cases included in the study.

Case n.	Tissue type	Diagnosis	Age (years)	Cause of death
1	Brain	ND	8-12 weeks	Miscarriage
2	Brain	ND	21	Non natural
3	Brain	ND	63	Non natural
4	Brain	ND	65	Non natural
5	Brain	ND	68	Non natural
6	Brain	ADI	> 70	AD
7	Brain	ADII	> 70	AD
8	Brain	ADIII	> 70	AD
9	Brain	ADIV	> 70	AD

ND: not determined. ADI, ADII, ADIII, ADIV: Alzheimer's diseases stages of Braak and Braak (see **Gil et al, 2017**).

Supplementary Table S2. Comparative analysis of nuclear tau epitopes in SK-N-BE cells and human hippocampal neurons across ages and AD stages.

	Tau epitopes				
Sample type	AT8	Tau-1	PHF1	T181	S262
SK-N-BE cells					
Proliferative	–	+	+	+	+
Differentiated	+	+	+	+	+
Human hippocampal tissue					
Fetus	60.1%	61.8%	65.8%	66.9%	63.1%
Young	67.8%	57.6%	57.6%	63.7%	57.3%
Senile (63y)	50.4%	ND	ND	ND	ND
Senile (65y)	50.7%	56.7%	52.4	58.3%	53.7
Senile (68y)	50.0%	ND	ND	ND	ND
AD-I	27.6%	54.5%	49.5%	55.0%	51.7%
AD-II	26.9%	ND	ND	ND	ND
AD-III	26.2%	ND	ND	ND	ND
AD-IV	21.6%	52.8%	46.6%	51.2%	45.2%

Notes:

For SK-N-BE cells, “+” indicates detectable nuclear localisation of the epitope; “–” indicates absence.

For human tissue, values represent the percentage of neurons positive for each epitope in the CA1 hippocampal region. ND, not determined.

This table summarizes the main findings for all analyzed nuclear tau epitopes under different experimental and pathological conditions.

Supplementary figures

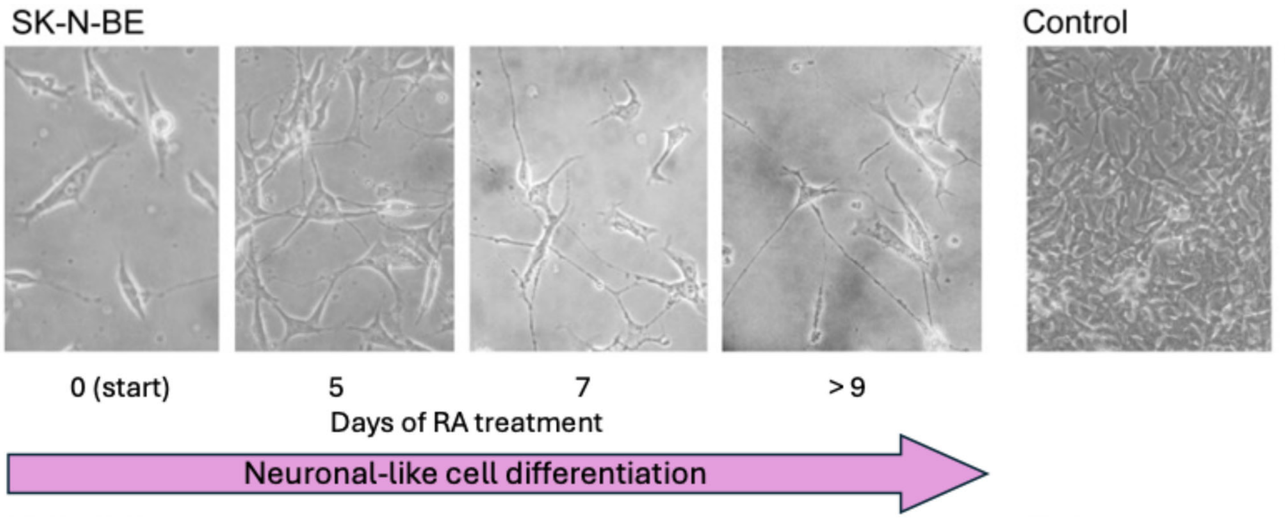

Figure S1. Differentiation of the SK-N-BE neuroblastoma cell line upon Retinoic Acid (RA) treatment. Cells were incubated with 10 µM RA and induced to differentiate into neuron-like cells, as described in the Materials and Methods section. Neurite-like processes became evident after a few days of RA exposure, with differentiation completed after 9–10 days of treatment.

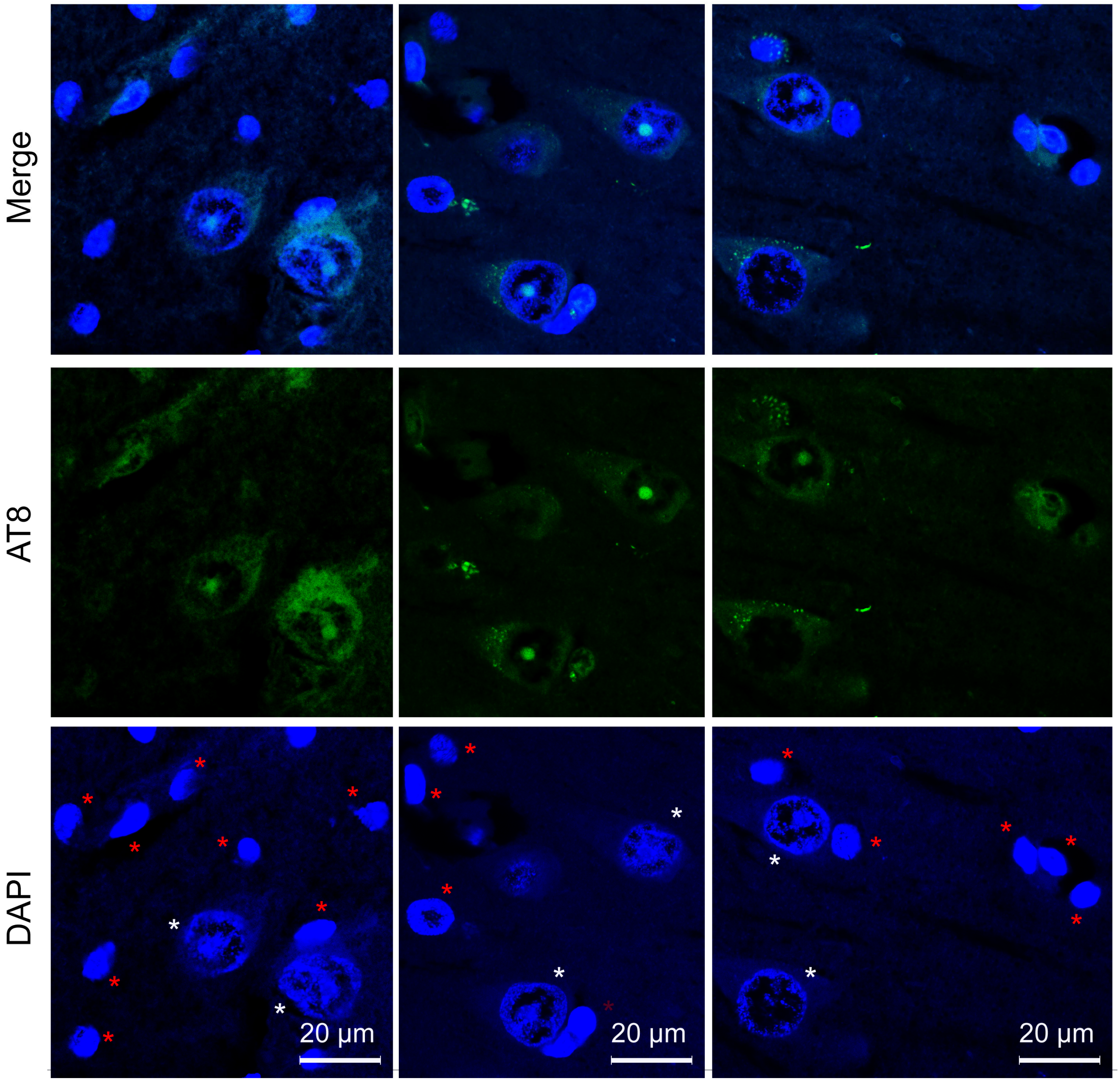

Figure S2. Identification of pyramidal neurons in the CA1 region. Pyramidal neurons were identified based on nuclear morphology revealed by DAPI staining (bottom panels). Characteristic features included: (i) large nuclei enriched in euchromatin, and (ii) a prominent, centrally located nucleolus (nuclei marked with white asterisks in the representative images). These criteria distinguish neurons from glial cells, which display smaller nuclei enriched in heterochromatin and typically lack a discernible nucleolus (nuclei marked with red asterisks). Green signals (middle panels) correspond to AT8 epitope immunodetection.

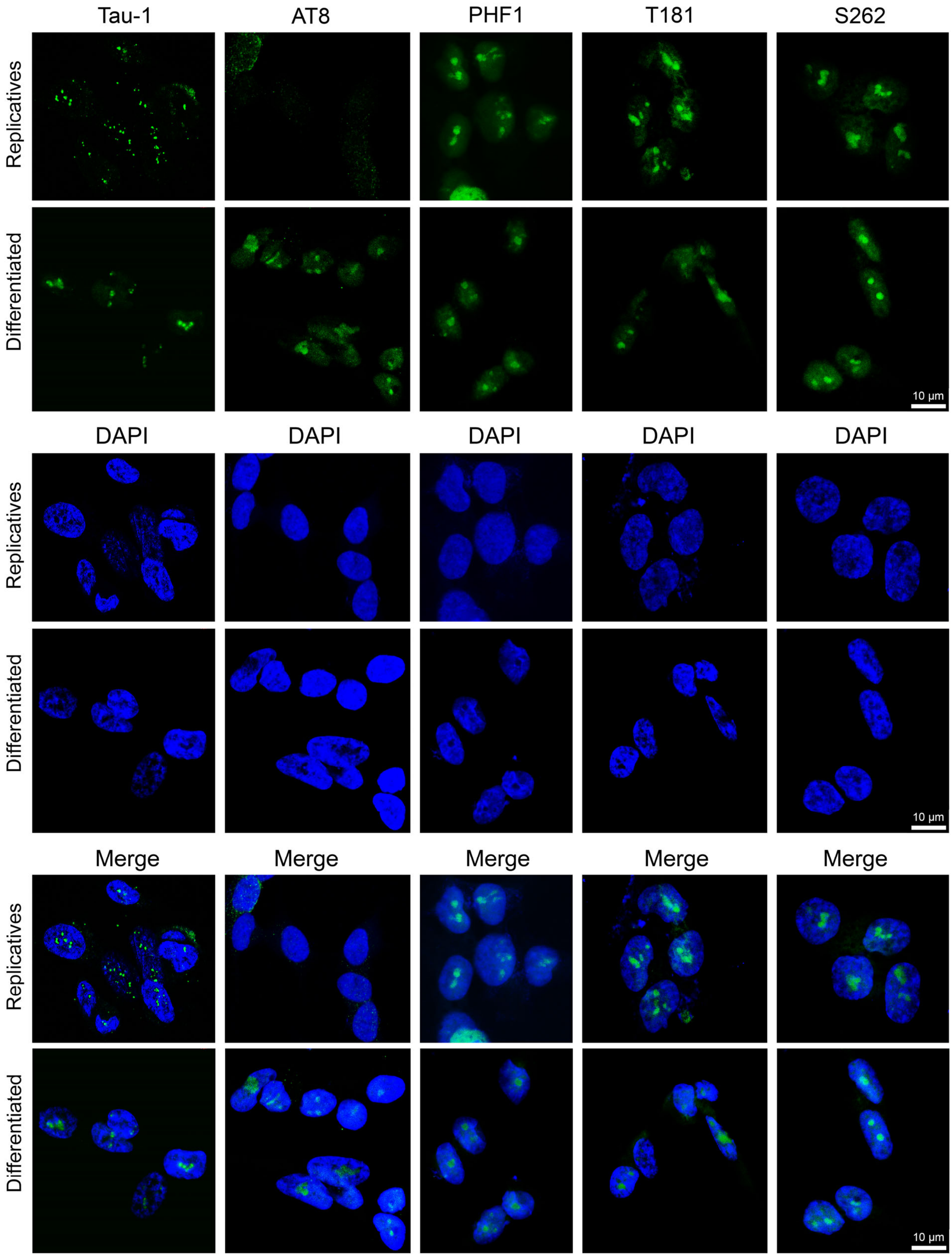

Figure S3. Visualisation of the green and blue channels corresponding to the images shown in Figure 1. Individual channels (green: Tau epitopes; blue: DAPI nuclear stain) and merged images are shown for each immunodetection presented in Figure 1 of the main text. Tau-1, AT8, PHF1, T181, and S262 epitopes were immunodetected in SK-N-BE cells under two conditions: replicative and differentiated cells. Scale bar: 10 µm (bottom right corner, applicable to all images).
